# Emotional release and physical symptom improvement: a qualitative analysis of self-reported outcomes and mechanisms in patients treated with neural therapy

**DOI:** 10.1186/s12906-018-2369-4

**Published:** 2018-11-27

**Authors:** Heidemarie Haller, Felix J. Saha, Barbara Ebner, Anna Kowoll, Dennis Anheyer, Gustav Dobos, Bettina Berger, Kyung-Eun Choi

**Affiliations:** 1 Department of Internal and Integrative Medicine, Kliniken Essen-Mitte, Faculty of Medicine, University of Duisburg-Essen, Essen, Germany; 2 Institute for Integrative Medicine, Department of Health, Witten/Herdecke University, Herdecke, Germany; 3 Institute for Medical Sociology, Health Services Research, and Rehabilitation Science (IMVR), Faculty of Human Sciences & Faculty of Medicine, University of Cologne, Cologne, Germany

**Keywords:** Neural therapy, Procaine, Chronic Disease, Body Image, Emotions, Qualitative Research

## Abstract

**Background:**

Neural Therapy (NT) is a common complementary treatment approach using injections with short-acting local anesthetics to treat pain and chronic diseases. However, little is known about the underlying mechanisms and the domains of treatment response. This study therefore analyzed patient experiences following NT injections with procaine.

**Methods:**

Maximum variation sampling was used to collect data from semi-structured interviews conducted with 22 hospital inpatients aged 59.6 ± 14.9 years (81.8% female). Each had multiple (9.4 ± 6.9) diagnoses. They were undergoing two weeks of integrative treatment, which included individualized NT. The interview data were analyzed in MAXQDA using qualitative content analysis.

**Results:**

With injection, patients first described local anesthetic effects including temporary blocking of pain and increased local warmth. Second, patients reported on vegetative reactions frequently leading to turmoil within the body like initial aggravation of existing symptoms or the appearance of new, concealed or phantom symptoms. This often required the need for rest to deal with the treatment stimulus. As a third step, many patients could gain physical and emotional release and relief in symptoms, mood and functioning. Emotional release was often accompanied by weeping and initially overwhelmed affected patients with dissociated memories. However, in cases where patients were able to experience those memories with a new distance, a fourth step of integration was achievable. It included reframing processes as well as a gain in pain perception and body-awareness. As a possible fifth step, patients experienced improved mood, increased pain acceptance and empowerment. Adverse events of NT included pain from the injections, vegetative complaints and emotional turmoil that lasted for minutes or hours, with a maximum of two days.

**Conclusions:**

Patients treated with procaine injections reported different psychophysiological outcomes contributing to the understanding of the mechanisms underlying NT. Further efficacy studies should separate specific NT from non-specific/placebo effects.

**Trial registration:**

DRKS00004567.

**Electronic supplementary material:**

The online version of this article (10.1186/s12906-018-2369-4) contains supplementary material, which is available to authorized users.

## Background

Neural Therapy (NT) uses injections of short-acting local anesthetics (mainly procaine) for diagnosis and treatment of chronic pain conditions and functional diseases [1]. According to Huneke, NT is classified into three main approaches. Firstly, local treatment techniques administer procaine directly or close to affected structures (e.g. infiltration into myofascial trigger points). Secondly, segmental techniques deliver procaine to structures innervated by the same spinal segment as the symptomatic area (e.g. dermatome quaddels, injections at muscles, joints and ganglia). Finally, interference field techniques infiltrate procaine into areas of subclinical chronic inflammation (often located in scars, fractures, teeth, sinuses and intestines) outside the respective anatomical segment [2–5]. The effects of treating interference fields were first described in 1940 in a patient with arthritic scapular pain, which directly decreased after procaine infiltration in an osteomyelitic leg scar, outlasting the duration of any expected pharmacological effect [3, 6]. NT is not only used for its potency in nerve-blocking effects, but also for its anti-inflammatory effects, which are believed to systematically modulate neuroplastic processes of peripheral sensitization, post-synaptic long-term potentiation, and pathological dysfunctions of the autonomic nervous system [7, 8]. Typical indications include chronic pain conditions, gastrointestinal disorders, rheumatoid and inflammatory diseases and functional cardiovascular syndromes. Contraindications are allergies to local anesthetics, coagulopathy, anticoagulation, and dermal infections. NT is widely used in Europe [9, 10], in Central and South America and the United States – sometimes being referred to as “nerve blocks” causing “regional anesthesia” [1].

Research into NT is limited. A 2005 Health Technology Assessment revealed evidence from case series and retrospective cohort data showing symptom improvement in up to 80% of 3812 treated patients, and preliminary data on cost-effectiveness in ambulatory care [11]. Further prospective observational trials showed effects of NT on subacute and chronic pain outcomes [12–15], impaired circulation [16], and safety of the applied injection techniques [17, 18]. Despite this, little is known about the underlying mechanisms of NT or the domains of treatment responses. In preparation for randomized controlled trials, this study aimed to investigate the effects and side effects of NT as perceived by chronically ill patients treated in the context of an internal and integrative medicine inpatient unit.

## Methods

This study was conducted in accordance with the Consolidated Criteria for Reporting Qualitative Research (COREQ) guidelines [19].

### Study design

The study was designed as a single center, qualitative interview study with post-intervention assessment. Prior to patient recruitment, the research protocol was approved by the Ethics Committee of the University of Duisburg-Essen, Germany (12–5208-BO), and registered at the WHO International Clinical Trials Registry Platform/German Clinical Trials Register (DRKS00004567). The study was conducted between November 2012 and November 2014 at the Department of Internal and Integrative Medicine, Kliniken Essen-Mitte, University of Duisburg-Essen, Essen, Germany.

### Sampling

Maximum variation sampling was used [20] to generate a heterogeneous sample of patients of different ages and sexes who reported varied effects from NT. Sample size was calculated up to 30 patients, with the intent of stopping recruitment when the interview data became saturated. Patients were invited to join the study by their treating physician, if they matched the following eligibility criteria: were aged 18 years or above, were being treated with NT during their inpatient treatment at the Department of Internal and Integrative Medicine, Kliniken Essen-Mitte, were German-speaking, and were willing to give their written informed consent for participation and publication of anonymized quotes. Patients were excluded in cases of severe comorbid mental illness or severe neurological disease.

### Intervention

During the two-week inpatient stay, patients received individualized integrative treatment including conventional as well as complementary therapies [21]. If indicated, NT was applied using a combination of local, segmental and interference field techniques with a maximum single dose of 25–30 ml of 1% procaine solution. The procaine injections were administrated in accordance with the patient diagnosis and reported symptoms and repeated as necessary [1]. All study treatments were performed by a male physician (FJS) with 18 years of experiences in the treatment and teaching of NT.

### Data assessment

The researchers used semi-structured interviews to collect the study data. The interview guide developed by the research group (HH, FJS, BB, KEC) was piloted with three patients receiving the described study treatment. The guide’s final version included both general and more detailed questions about the patients’ treatment expectations, their physical and psychological responses to the NT, the effects on quality of life, and treatment safety (Additional file 1). Further open-ended questions on each of the above topics could be used if necessary. The study interviews were conducted face-to-face by two female psychologists (HH, KEC), both of whom had advanced training in qualitative research methods. Neither of the interviewers were involved in delivering the study treatments. Each interview started by introducing the interviewer and outlining their reasons for engaging in the research explaining the study’s aims. The interviews lasted on average 19.5 min, generating a total of 7.13 h of data. All interviews were audio-recorded, professionally transcribed verbatim and pseudonymized. The interviewers’ field notes and patient diagnoses were then added to the pseudonymized transcripts.

### Data analysis

The interviews with the original German language text were coded independently by two pairs of coders (AK, BE and HH, KEC) using MAXQDA software (Version 11.0.9, VERBI). The resulting codes were summarized to minor and major themes and paraphrased by an interdisciplinary group consisting of a physician (FJS), two medical students (AK, BE), two psychologists (HH, KEC), and a cultural scientist (BB) using inductive qualitative content analysis techniques [22]. Respective patient quotes were identified using the patient study number and the number of the paragraph within each transcript. For publication purposes, selected quotes were professionally translated into English.

## Results

### Sample characteristics

The final study sample consisted of 22 inpatients (81.8% female) aged 59.6 ± 14.9 years. No patient refused to participate or withdrew consent for any reason. Most patients had multiple morbidities, with an average of 9.4 ± 6.9 diagnoses each. The main conditions for which they were receiving treatment included polyarthritis, osteoarthritis, spondylosis, radiculitis, fibromyalgia, chronic neck pain, tension headaches and migraine, irritable bowel syndrome, sinusitis, fatigue, recurrent depressive episodes and pain after surgery for metastatic breast cancer. During their inpatient stay, patients received on average 1.32 ± 0.95 NT treatments. Individual treatment characteristics can be found in the Additional file 2.

### Qualitative content analysis

The qualitative data analysis process, described above, generated 278 codes, grouped hierarchically under 5 major and 19 minor themes (Table 1):

**Table 1:** Major and minor themes derived from the qualitative content analysis

Major themes	Minor themes
1. Expectations and premises	Hopes and fears Safe and trustworthy setting
2. Local and systemic bodily responses	Immediate anesthetic effects Vegetative responses Need for rest Initial symptom aggravation Pain and symptom alleviation Reintegrated body image and increased awareness Improvement in daily functioning
3. Emotional release and contentment	Emotional release reactions Integration of dissociated memories Cognitive reframing Bodily responses Improved mood and enjoyment of life Retrieving personal agency
4. Adverse events	Pain from the injection Vegetative complaints Emotional turmoil
5. Patients’ conclusions	Significance of the treatment effects

#### Theme 1: Expectations and premises

##### Hopes and fears

Patients reported hope of improvement as well as attitudes of ‘wait and see’ and ‘nothing to lose’. Several patients also emphasize fears about injections and possible treatment side effects.

*- “I considered this as a chance. I thought: What do I have to lose? I have nothing to lose.” (13|58)*

*- “I was really worried. I don’t want to leave this hospital in a wheelchair, I told the doctor. But he reassured me and I’m very thankful that I could ask all of my questions.” (10|62)*

##### Safe and trustworthy setting

Patients stated a trustful relation to the doctor as most important to feel safe and engage in the invasive NT treatment.

*- “You need trust. I would not allow anyone to use such long needles on such sensitive body parts.” (20|93)*

*- “The safe environment here, which I did not have outside, to allow yourself to fall.” (05|110)*

#### Theme 2: Local and systemic bodily responses

##### Immediate anesthetic effects

Following the injections, patients described a number of local anesthetic effects, including the disappearance of pain, sensations of diffuse pressure and improved circulation adjacent the treated vessels.

*- “Well, you feel a little prick and later on a diffuse feeling of pressure.” (10|44)*

*- “My pain was suddenly blown away. […] And it became warm, right up to the knee. And the next time, right up to the foot.” (16|43–46)*

##### Vegetative responses

Patients frequently reported a range of vagotonic/sympathicolytic reactions following their NT. These included nausea and dizziness, as well as more pleasant feelings of relaxation, ease, inner peace and emptiness. Some patients noted that the latter reminded them of the altered states of consciousness that they have experienced during meditation. On a physical level, patients also described sensations of body warmth and surprising changes to their nasal secretion, urination and menstruation.

*- “I got a little dizzy at first, but only for a short while, then I felt warmth spreading out into my feet. A very comfortable feeling.” (14|42)*

*- “This was such an exhilarating feeling. There was nothing: you felt nothing, you thought nothing, nothing at all.” (13|30)*

*- “As if I was on a beautiful cloud. Such a calm, content and decelerated feeling. [...] I was very close to myself, very focused. The view goes deeply inwards. Similar to what happens when I practice Yoga.” (20|41,45)*

*- “During the injection, I noticed that my eyes were welling up and that my nose was running. That did not happen for the last 20 years! I was so happy at having a runny nose.” (07|47)*

*- “I had to go to the toilet about five times that night, passing water.” (17|56)*

*- “My periods started, which shouldn’t actually be possible, because I haven’t had any periods since I got my coil put in, two years ago.” (21|66)*

##### Need for rest

Some patients found their NT treatments exhausting, both physically and mentally, at times requesting additional time to deal with the treatment stimulus.

*- “Afterwards, I felt so tired but in a good mood because something was moving.”(03|55)*

- “I wanted to be alone. Walking through the park. That made me feel good.” (22|65)

##### Initial symptom aggravation

In addition to the above, some patients found that the NT treatment led to a temporary aggravation of their existing symptoms or the appearance of new, concealed or phantom symptoms; the latter were usually seen as harmless systemic responses.

*- “My upper back has improved but the pain of my lower back has increased.” (19|91)*

*- “And then, the teeth that I had had taken out started to hurt. [...] Isn’t that a sign that my whole body responded?!” (14|42,52)*

##### Pain and symptom alleviation

Patients cited pain relief as the main effect of NT, alongside a softening of their scar tissue and a decrease in their muscle tension. Patients reported that most of their other conditions also improved post-treatment although, for some, this relief was brief.

*- “At times, I had pain everywhere in my body. Every step hurt. And all of a sudden I can walk without any pain in my legs. So far, the pain has not returned. I am totally excited.” (21|64)*

*- “I have had more solid bowel movements for two days now. This is a very rapid success, considering that I’ve had severe, debilitating diarrhea for nearly nine months.” (20|73)*

*- “The next morning I thought: My hands - nothing hurts! I wasn’t sure; was that a dream? Was it real? How long would it last? Would it be gone by tomorrow? But it lasted until today. […] I still have pain in my back, although I’ve had two injections right here, but the tingling in my legs, the electrical ‘prickling’, is gone.” (13|34–38)*

##### Reintegrated body image and increased bodily awareness

Patients reported that their treatments had led them to regain sensitivity in parts of their bodies that had previously been impaired, describing the treated areas as generally more ‘vivid’. Their treatment also helped them to reintegrate parts of their bodies from which they had formerly felt alienated so that they felt more ‘whole’ again. These outcomes enhanced the patients’ bodily awareness, increasing their confidence in their personal feelings and intuitions.

*- “My foot was limp. You gave a try, you tried to move the foot, but nothing happened. And, at the moment that I had the injection, it felt as if life re-entered my foot.” (18|99)*

*- “When the arthritis worsened, and got more painful, I felt as if my leg didn’t belong to me. Like a foreign object, a peg leg. And after the injection, my leg felt more vivid. It was mine. […] It’s great, growing back together again.” (16|45,97)*

*- “That supports me in listening even more to my gut feelings. [...] And I believe that I might be able to get much closer to my inner self, and trust more in what I really feel.” (20|67)*

##### Improvement in daily functioning

Improving symptoms made patients feel notably younger. Many voiced excitement that their horizons have now broadened, in terms of their mobility and daily functioning; this occurred often after a long history of illness and suffering.

*- “When you’ve got so many different places that hurt, you feel like you’re in a vice. As if my body was eighty years old. [...]. Now, I feel considerably younger!” (18|137)*

*- “Afterwards, I felt as if I’d been in the fountain of youth [...]. And all the others said: Wow! You’re looking really great!” (03|63)*

*- “Joy! Joy! […] I didn’t need my crutches anymore! I can’t believe it, after three and a half years!” (04|83)*

*- “I’ve been discharged from medical rehabilitation, due to pain, and unable to work. Now, I have the courage to say, okay, I can imagine working for eight hours.” (13|50)*

#### Theme 3: Emotional release and contentment

##### Emotional release reactions

Seven patients found that their injections triggered memories, overwhelming them with suppressed emotions. They reported recalling images at a high frame rate, experiencing them as uncontrollable and/or initially confusing; at times this was accompanied by shivering or nausea. All of these patients did not evaluate the release of emotions as frightening, but rather as a relief.

*- “Images flashed through my head, from right to left. I felt pretty confused.” (06|50)*

*- “The injection made me think ‘I have to surrender’. I’ve had such a lump in my throat. […] And then, like a tornado, like a volcano, ‘I have to cry’. I’ve only ever seen pictures of my father. He died 49 years ago. I never said goodbye to him.” (02|40,56)*

*- “When I got the injection, it all spouted out of me. Suddenly, I saw images, memories resurfaced, I started to cry bitterly, I shivered. [...] I couldn’t control it at all. All those emotions came up to the surface again. I’m glad that it happened, because now it’s gone. It wasn’t threatening; in fact it was a relief.” (21|24,65)*

##### Integration of dissociated memories

Allowing post-treatment emotional release enabled many patients to see their personal memories from an increased distance, helping them to reevaluate and reintegrate their memories into new emotional contexts. This led patients to better acceptance and reconciliation of their personal history.

*- “And then I could allow that, tick, tick, tick, images from my memory popped up, like in a movie. I went through my entire tonsillectomy again. But this time, I saw the scene more as if from above. At the end, I felt that it was okay. […] That’s the way it happened. It wasn’t as frightening as the real surgery.” (01|52–54)*

*- “Well, it was like a film running backwards. Like watching your life in reverse. [...] It was very realistic, or maybe not realistic, but like a critical view of something that you can’t change, anyway.” (03|135–137)*

##### Cognitive reframing

The new associations that patients made between their memories and emotions led them to understand the context and function of their illnesses in greater depth.

*- “I only knew that I had this deep sadness inside, which I couldn’t identify. I thought that I would’ve processed the miscarriage; that I could deal with it, but that wasn’t true. That wasn’t obvious to me. Not at all.” (21|34)*

##### Bodily responses

Some patients linked their emotional release directly to improvements in their physical symptoms and body perception.

*- “When I got the injection, suddenly, the images came back, everything that happened that day. All the feelings came back. […] And I cried, bitterly, until it stopped. And the next day, I felt such a release, as if a belt around my heart has split. […] I feel like I can breathe again, that the sadness is gone. With one treatment! And the pain in my hands has decreased. And the pain in my shoulders as well.” (21|28)*

##### Improved mood and enjoyment of life

Other patients did not report emotional release during their NT treatment, but found themselves feeling surprisingly happy and lighthearted, when it had finished. Reductions in their pain levels also led to a growing confidence and a new enjoyment of life after the lengthy periods that many had spent fighting against illness.

*- “The next morning I woke up and thought that something is different. Although the weather was actually cloudy, to me all the world seemed bright.” (11|27)*

*- “I feel physically and mentally relieved. It’s really depressing having pain all the time, but you don’t want give up. You tackle the problem, but it’s always a struggle, a fight. And now, there’s less fighting and that’s great.” (06|120)*

##### Retrieving personal agency

Some patients also reported on increased feelings of personal control over their everyday lives, increased engagement in active decision-making and greater feelings of empowerment and autonomy.

*- “Before the therapy, I cried a lot, because nothing seemed to work anymore. Now, I feel stronger, because the load is gone, this burdensome situation.” (19|77)*

*- “I looked back carefully, once again, and decided to finish the whole thing off. It was so many years ago. This might be a new beginning for me.” (03|105)*

#### Theme 4: Adverse events

##### Pain from the injection

The main adverse event that patients experienced was pain at the time of their injections. They generally tolerated this because they expected the injections to improve their symptoms.

*- “The initial prick hurts and it continues to hurt until the needle has found its position. Then, the injection of the procaine causes a feeling of pressure. Sometimes I need to say ‘Stop - slower, please’, until the anesthesia occurs. Then it’s okay. That’s always a challenge.” (20|31)*

##### Vegetative complaints

Other adverse events included vertigo, hot flashes, tiredness, headaches and exhaustion. Most of these outcomes were gone within minutes or hours, although some lasted for up to two days.

*- “And then I just felt sick. I thought I have to throw up. I felt dizzy.” (02|38)*

*- “During the injection, I had hot flashes, which disappeared quickly. [...] Afterwards, I did feel quite exhausted, and sometimes a bit dazed, the closer the injections got to my head.”(19|35,27)*

##### Emotional turmoil

Some patients reported nightmares, the need to cry and/or the relive of previously traumatic events. These events unexpectedly faded away as quickly as they came.

*- “I had nightmares, really bad nightmares. But they stopped as suddenly as they appeared.” (06|104)*

*- “These memories appeared more frequently over the course of the next day and the next afternoon. They were quite intense.” (05|67)*

#### Theme 5: Patients’ conclusions

##### Significance of the treatment effects

Most patients reported a temporary or sustained reduction in their symptoms, with many experiencing emotional relief and improved well-being. Three of the study patients said that NT had no effect on their pain intensity and two others that it did not affect their mood.

Patients attributed a range of local and systemic effects directly to their NT treatment. The integrative inpatient treatment setting also was perceived as supportive. Most patients reported that, based on their current experiences of NT, they would use it again.

*- “Quality of life. Absolutely! Before the Neural Therapy, I managed my life alongside the pain. You took more pills, you took part, also laughed with other people, but you were strained all the time. Now my pain has got less, I’ve realized how relieved I am and how great it is.” (06|118)*

*- “When the pain in my knee cartilage reappeared, I was very disappointed.” (16|107)*

*- “[There was] no mood brightening, or anything like that. It was all quite normal.” (10|65)*

*- “Here, Neural Therapy is embedded in a larger concept. This has led to more stability. Together with other tools that I use, I think, this has led to a more long-lasting effect.” (16|62)*

*- “If I feel worse again, I would consider using Neural Therapy again.” (13|66)*

## Discussion

### Summary of evidence

Patients treated with NT reported local anesthetic effects and systemic bodily responses resulting in the reduction of their pain and other symptoms, feelings of emotional release/relief and improvements in their general well-being in most cases. They perceived the treated areas generally as more vivid and better reintegrated into their body image. Improvements in daily functioning made patients feeling younger and enhanced their quality of life. NT injections also triggered states of altered consciousness, sometimes accompanied by the emergence of memories and the release of emotions. These processes of recall and release enabled patients to view their memories more from a perspective of a distant observer, helping them to reevaluate and reintegrate their memories into a new emotional context. This outcome led to emotional relief, cognitive reframing, pain reduction and a sense of empowerment. Other patients did not report distinct emotional events, but were surprised by post-treatment feelings of happiness, confidence and regained enjoyment of life. Adverse events included pain due to the injections, vegetative complaints and emotional turmoil that generally lasted for minutes or hours with a maximum of two days. In relation to the desired treatment effects, patients assessed these side effects as negligible.

### A theory of action

The above findings contribute to a deeper understanding of the mechanisms underpinning NT. They suggest that health improvement may be theorized as a process consisting of the following steps:

1. Temporary Block: Local anesthetics temporarily block pain transmission that patients perceive as the immediate disappearance of their pain [7]; this often induce euphoric states that patients describe variously as eased, spaced-out or dazed.
2. Turmoil: Besides interrupting the pain, NT appears to stimulate responses of the autonomic nervous system, frequently leading to turmoil within the body. Study patients described sensations of dizziness at this stage, and the need to rest in order to deal with the treatment stimulus. Their existing symptoms could be temporarily aggravated and new or phantom symptoms could occur. NT theory generally views such sensations as positive treatment responses. Reverse/reaction phenomena, for example, are defined as the worsening of treated symptoms shortly after segmental injection [23]. Other complementary therapies have also been found to worsen patient symptoms initially [24–26]. Retrograde phenomena flowing segmental treatment, on the other hand, are defined as the development of pain in a related interference field that had previously been clinically silent [1]. This may explain the report of new symptoms not apparently linked to their primary pain. Turmoil of emotions, moreover, was reported by several patients, seemingly associated with injections into scar tissue. Such reactions are also known from the application of manual treatments like massage or Craniosacral Therapy [24, 27]. Emotions were partly linked to former stressful life events, in which patients had to fight or freeze and were unable to express their emotions. Such experiences can lead to a chronically increased sympathetic tone and muscle tension whilst the emotional load subsides [28]. Later relaxation of the tissue tension, in reverse, can lead the suppressed emotions to resurface, triggered by the somatic markers with which they are still associated [28, 29].
3. Release and Relief: Subsequent to the experience of turmoil, which seemed to be a sufficient but not a necessary condition, patients could perceive physical and emotional release as well as relief in symptoms, mood and improved daily functioning. On a bio-physiological level, this link is explainable by the pharmacology of local anesthetics. Beside the reversible block of voltage-gated Na^+^-channels, procaine is known to modulate the inflammatory response and sympathetic nervous system activity by reducing the secretion of proinflammatory cytokines, inhibiting the signal transduction via G-protein coupled receptors and muscarinic m1/m3 receptors [30–32], and enhancing local endocannabinoid levels by inhibiting fatty acid amide hydrolase expression [33]. This increases local perfusion and relieves tension within the fascial and muscle tissue. The benefits of emotional release might be explained by Porges’ Polyvagal Theory. In addition to fighting and freezing reactions, which are mediated by the sympathetic and parasympathetic (unmyelinated dorsal vagus) nervous system, Porges hypothesizes that a second parasympathetic mode (myelinated ventral vagus) fosters oxytocin secretion, social communication, self-soothing/calming, and inhibits sympathetic-adrenal arousal in safe environments [34]. Research findings for manual therapies [35, 36] and acupuncture [37] may also apply to NT that may similarly increase vagal activity and activate the amygdala, hypothalamus and anterior cingulate cortex, which are regions known to regulate stress and emotion. Generating a safe therapeutic environment also seems to make a difference between emotional reorganization and retraumatization [38].
4. Integration: Symptom relief can lead to consolidation and integration mechanisms, which may include improvements in pain perception and body/self-awareness [39] as well as the reintegration and reevaluation of dissociated memories and disease beliefs [40]. Both basic [41] and clinical research [42] agree that patients often have to revisit the emotionally distressing memory trace, allow the physiological arousal and following relaxation, before they can reevaluate and reintegrate their memories.
5. Empowerment: As a result of their NT treatments, the study patients reported that they felt empowered and better prepared to engage in a more active life.

### Limitations

This study has a number of potential limitations. Firstly, the inpatient setting might limit the generalizability of the findings. Although only quotes that referred clearly to NT were entered into the qualitative content analysis, it remains possible that study outcomes are not solely due to NT treatment but are due to combination with other inpatient treatments. Secondly, the study data were collected from participants during their inpatient stay. Thus, the data only reflect experiences and perceptions immediately after or a few days after their NT treatments. Questions about the possible persistence of any of the observed changes must therefore remain unanswered. Finally, it should be noted that conclusions drawn from qualitative data, even if maximum variation sampling was used, are neither representative nor reveal causal relations, as we could not control for placebo and non-specific treatment effects.

### Implications for further research

Further studies should address the link between emotional release and physical relief phenomena. In addition, it should be investigated whether the observed effects are specific to NT in contrast to sham (efficacy research) or similar to other complementary or psychological treatment techniques (comparative effectiveness) [43]. Qualitative designs as well as rigorous randomized controlled trials with adequate sample sizes and standardized outcome measures are necessary; these should not be limited to a physical symptom level but include tools to quantify effects of release, integration and empowerment as well.

## Conclusions

Patients treated with procaine injections reported different psychophysiological outcomes ranging from improvements in pain and chronic symptoms to enhanced daily functioning and mental quality of life. In cases where usual or local treatment approaches are not effective, expanded segmental and interference field techniques might offer a promising treatment option. Further quantitative trials should answer the question whether the observed effects are specific to NT or a consequence of unspecific mechanisms associated with the patients’ expectations, therapist attention, or other setting conditions.

## Additional files

Additional file 1: Semi-structured interview guide. (DOCX 17 kb)

Additional file 2: Individual NT treatments. (DOCX 19 kb)
