# Supplementary material for: Emotional release and physical symptom improvement: a qualitative analysis of self-reported outcomes and mechanisms in patients treated with neural therapy
Source: BMC Complement Altern Med. 2018 Nov 27;18:311. doi: 10.1186/s12906-018-2369-4 (PMC6258402; doi:10.1186/s12906-018-2369-4)
Supplement: Supplementary file 1 — Semi-structured interview guide. (DOCX 17 kb) [file 12906_2018_2369_MOESM1_ESM.docx]

Additional file 1. Semi-structured interview guide

| **Topic** | **Main questions** | **Further questions** |
| --- | --- | --- |
| Initiation | - To what extent did you have expectations or concerns about Neural Therapy before your treatment? - Which symptoms were treated with Neural Therapy? | - How did you experience the treating doctor? - Can you please describe the course of your treatment in more detail? |
| Physical responses to treatment | - How did you perceive the treated area straight after your injection? - To what extent did you experience further bodily responses to the treatment? | - To what extent did these reactions change after the treatment? - To what extent did you experience effects on your circulation, body temperature, digestion, or sleep? |
| Psychological responses to treatment | - To what extent did you experience changes in your mood during or after your Neural Therapy treatment? | - To what extent did you experience strong emotions such as crying, fear, or happiness? |
| Effects on quality of life | - To what extent did you experience changes in your daily functioning or quality of life? | - How long did these changes last? - To what extent do you think these changes occur due to Neural Therapy? |
| Safety | - To what extent did you perceive Neural Therapy as unpleasant or painful? | - How would you assess these side effects in relation to the desired treatment effects? |
