# Supplementary material for: Emotional release and physical symptom improvement: a qualitative analysis of self-reported outcomes and mechanisms in patients treated with neural therapy
Source: BMC Complement Altern Med. 2018 Nov 27;18:311. doi: 10.1186/s12906-018-2369-4 (PMC6258402; doi:10.1186/s12906-018-2369-4)
Supplement: Supplementary file 2 — Individual NT treatments. (DOCX 19 kb) [file 12906_2018_2369_MOESM2_ESM.docx]

Additional file 2. Individual NT treatments

| **Patient study number** | **Main diagnosis** | **Number of additional diagnoses** | **Number of Neural Therapy treatments** | **Treated areas/points** |
| --- | --- | --- | --- | --- |
| 1 | Chronic polyarthritis | 3 | 1 | Tonsils, plexus utero-vaginalis, plexus hypogastricus inferior, navel, sinuses, thyroid gland, teeth 36,37 |
| 2 | Chronic neck pain | 11 | 2 | 1. Treatment: plexus uterovaginalis, epidural-sacral injection, navel  2. Treatment: facet joints L3/L4, L4/L5 |
| 3 | Peroneal paralysis | 25 | 1 | Scar of tonsillectomy, sinuses, right ganglion oticum, right ganglion pterygopalatinum, teeth, right ear |
| 4 | Chronic neck pain | 6 | 1 | Sacroiliac joint, right hip, right trochanter major |
| 5 | Fibromyalgia | 5 | 1 | Scar left leg, sinuses, thyroid gland, abdominal scars, plexus hypogastricus inferior |
| 6 | Fibromyalgia | 15 | 1 | Trapezius muscle, quaddels cervical and lumbar spine, facet joint L5/right, scars at the neck and back, popliteal fossa, nervus occipitalis major, processus spinosus C7, teeth, tonsils, right elbow |
| 7 | Spondylosis with lumbar radiculopathy | 20 | 1 | Sinuses, tonsils, epidural-sacral injection, rigth heel spur, abdominal scar |
| 8 | Fibromyalgia | 5 | 1 | Thyroid gland, plexus hypogastricus inferior, navel, scars, left ankle joint |
| 9 | Migraine | 1 | 1 | Abdominal scar, plexus hypogastricus inferior |
| 10 | Lumbar spondylosis | 10 | 1 | Epidural sacral injection, processus spinosus C2, teeth, tonsils, lumbar spine |
| 11 | Lumbar/sacral radiculopathy | 9 | 2 | 1. Treatment: navel, abdominal scar  2. Treatment: navel, abdominal and lumbar scars |
| 12 | Crohn's disease | 6 | 1 | Abdominal scar, epidural-sacral injection, plexus uterovaginalis |
| 13 | Fibromyalgia | 5 | 1 | Sinuses, teeth, scar of the tonsillectomy, thyroid gland, Plexus hypogastricus inferior, abdominal scar, navel, sacroiliac joint |
| 14 | Chronic pain syndrome | 3 | 1 | Ganglion sphenopalatinum, sinuses, ganglion oticum |
| 15 | Ulcerative colitis | 2 | 1 | Teeth, tonsils, navel, Vogler-points, acupuncture points: St 25, 27; Sp 13, 15, CV 4, 12, 15 |
| 16 | Generalized primary osteoarthritis | 9 | 3 | 1. Treatment: scars right leg  2. Treatment: right arteria femoralis, right knee  3. Treatment: scars right leg |
| 17 | Fibromyalgia | 9 | 1 | Right ganglion stellatum, thyroid gland, tonsils, right shoulder |
| 18 | Tension headache | 6 | 4 | 1. Treatment: epidural-sacral injection, scars, left arteria femoralis  2. Treatment: arteria dorsalis pedis, tibialis posterior and arteria poplitea.  3. Treatment: right ganglion cervicale superius  4. Treatment: left ganglion cervicale superius |
| 19 | Breast cancer | 0 | 1 | Thoracic scars, ganglion stellatum, facet joints D3-5 |
| 20 | Breast cancer | 2 | 1 | Thyroid gland, plexus uterovaginalis, ganglion coeliacum, thoracic scars, abdominal scars, facet joints D4-8 |
| 21 | Fibromyalgia | 8 | 1 | Sinuses, thyroid gland, teeth, plexus uterovaginalis |
| 22 | Hip osteoarthritis | 24 | 2 | 1. Treatment: sinuses, teeth, tonsils, scar in the face, thyroid gland, abdominal scars, navel,  2. Treatment: acupuncture points Lu 3, St 30, Ki 11, CV 2, left nervus occipitalis minor et major, facet joints D 3/4, epidural-sacral injection, processus spinosus C 2 |
